# Activation of the NLRP3 inflammasome and elevation of interleukin-1β secretion in infection by sever fever with thrombocytopenia syndrome virus

**DOI:** 10.1038/s41598-022-06229-0

**Published:** 2022-02-16

**Authors:** Zhifeng Li, Jianli Hu, Changjun Bao, Chengfeng Gao, Nan Zhang, Carol J. Cardona, Zheng Xing

**Affiliations:** 1 Medical School and Jiangsu Provincial Key Laboratory of Medicine, Nanjing University, Nanjing, China; 2 Jiangsu Provincial Center for Disease Prevention and Control, Nanjing, 210009 China; 3 Department of Veterinary Biomedical Sciences, College of Veterinary Medicine, University of Minnesota at Twin Cities, 300D Veterinary Science Building, 1971 Commonwealth Avenue, Saint Paul, MN 55108 USA

**Keywords:** Viral infection, Virology

## Abstract

Severe fever with thrombocytopenia syndrome virus (SFTSV) is an emerging *phlebovirus* that causes a hemorrhagic fever known as the severe fever with thrombocytopenia syndrome (SFTS). Inflammasomes are a molecular platform that are assembled to process pro-caspase 1 and subsequently promote secretion of interleukin (IL)-1β/IL-18 for proinflammatory responses induced upon infection. We hypothesize that inflammasome activation and pyroptosis induced in SFTS results in elevated levels of IL-1β/IL-18 responsible for high fever and hemorrhage in the host, characteristic of SFTS. Here we report that IL-1β secretion was elevated in SFTS patients and infected mice and IL-1β levels appeared to be reversibly associated to disease severity and viral load in patients’ blood. Increased caspase-1 activation, IL-1β/IL-18 secretion, cell death, and processing of gasdermin D were detected, indicating that pyroptosis was induced in SFTSV-infected human peripheral blood monocytes (PBMCs). To characterize the mechanism of pyroptosis induction, we knocked down several NOD-like receptors (NLRs) with respective shRNAs in PBMCs and showed that the NLR family pyrin domain containing 3 (NLRP3) inflammasome was critical for processing pro-caspase-1 and pro-IL-1β. Our data with specific inhibitors for NLRP3 and caspase-1 further showed that activation of the NLRP3 inflammasome was key to caspase-1 activation and IL-1β secretion which may be inhibitory to viral replication in PBMCs infected with SFTSV. The findings in this study suggest that the activation of the NLPR3 inflammasome and pyroptosis, leading to IL-1β/IL-18 secretion during the SFTSV infection, could play important roles in viral pathogenesis and host protection. Pyroptosis as part of innate immunity might be essential in proinflammatory responses and pathogenicty in humans infected with this novel phlebovirus.

## Introduction

Severe fever with thrombocytopenia syndrome virus (SFTSV) is a tick-borne *phlebovirus* in the family *Phenuiviridae*, which causes severe fever with thrombocytopenia syndrome (SFTS), a fatal hemorrhagic fever disease endemic in East Asia^1,2^. The disease emerged in 2007 and was characterized by high fever, a drastic reduction of platelets and leukocytes in hosts which could result in multi-organ failure and hemorrhage in severe cases with mortality up to 12%^3–6^. Over 13,000 human cases have been reported in 23 Chinese provinces in the past decade^3,7^. SFTS has also been reported in Korea and Japan since 2012 and 2014, respectively, with cases from which virus was isolated^8,9^. But as shown in a retrospective study the earliest SFTS cases might be traced back in 2005 in Japan^10^.

Host innate immunity is the first line of defense to detect viral infection through the recognition of pathogen molecular patterns^11^. The best-characterized viral sensors are pattern-recognition receptors, including Toll-like receptors, RIG-I-like receptors, NOD-like receptors (NLRs), and C-type lectin receptors^12–14^. The NLRs are involved in the assembly of large protein complexes known as inflammasomes, which are critical in the innate immune response to pathogens. NLRs are composed of a conserved NAIP, CIITA, HET-E and TP1 domain (NACHT) and up to three other characteristic domains including a C-terminal leucine-rich repeat (LRR), and an N-terminal caspase recruitment domain (CARD), pyrin domain (PYD), or baculoviral inhibitor of apoptosis repeat (BIR). So far, at least 23 NLR genes have been identified in humans. A subset of NLRs, including NLRP1, NLRP3 and NLRC4, and a non-NLR absent in melanoma-2 (AIM2) with a pyrin and a HIN-200 domain, are often considered to contribute to pathogen clearance by mediating activation of pro-caspase-1^14,15^. Currently, the NLRP3 inflammasome is the most thoroughly studied inflammatory complex, consists of NLRP3 as a cytoplasmic sensor molecule, the apoptosis-associated speck-like protein containing caspase recruitment domain (ASC) as the adaptor protein, and the effector protein, pro-caspase-1. Upon activation, the inflammasome is assembled and promotes pro-caspase-1 self-cleavage to generate the active subunits p20, leading to the maturation and secretion of IL-1β and IL-18^14,16^. IL-1β and IL-18 are deemed to play crucial roles in proinflammatory responses by increasing vascular permeability via damaging vessel endothelial cells, facilitating neutrophil and lymphocyte recruitment to the site of infection, and promoting adaptive immune responses by inducing the expression of immunity associated genes^12,16^. Earlier studies revealed that SFTSV infection in humans induced expression of IL-1β, a proinflammatory cytokine, which was in significantly higher levels in the sera of severely and fatally affected patients than it was in mildly ill patients, indicating that IL-1β might contribute to a cytokine storm and deteriorating pathogenicity^17^. Despite this, there was a report that showed higher serum IL-1β levels in SFTSV-infected individuals was associated with lower viral loads in patient tissues or organs^18^. Studies that demonstrate the precise mechanism that regulates the expression and secretion of IL-1β in SFTS patients are limited.

In this report we attempt to further our understanding of IL-1β in viral pathogenesis during SFTSV infection. We analyzed the induction of IL-1β in SFTS patients and mice infected with SFTSV and our data revealed a mechanism by which an NLRP3 inflammasome was activated, resulting in the processing of pro-caspase-1 and secretion of IL-1β/IL-18 in human PBMCs. We further showed that SFTSV induced IL-1β secretion and pyroptosis which might restrict viral replication in cell cultures and mice, suggesting that the NLRP3 inflammasome may exert antiviral and host protective activities against SFTSV.

## Materials and methods

### Clinical specimens and blood samples

Sera of SFTSV-infected patients in the acute phase of the disease (n = 69) and healthy individuals (n = 17) were collected at the Provincial Center for Disease Control and Prevention of Jiangsu, Nanjing. Blood samples of healthy donors were randomly collected from the Nanjing Blood Center. To isolate PBMCs, whole blood was diluted in RPMI-1640 (Invitrogen, Carlsbad, CA) and PBMCs were separated in 15 ml centrifuge tube containing 5 ml lymphocyte separation medium (#50494) (MP Biomedicals, Santa Ana, CA) for centrifugation at 800×*g* for 25 min at room temperature (RT). The cell layer, rich in PBMCs, was transferred to a new centrifuge tube and cells were further diluted with RPMI-1640. Any red blood cells remaining in the PBMC fraction were removed using a red blood cell lysis buffer (Sigma-Aldrich, St. Louis, MO). Purified PBMCs were washed three times using phosphate-buffered saline (PBS) and pelleted at 800×*g* for 10 min. Monocytes were enriched by adherence to the plate (2 h at 37 °C) in phenol-red-free Dulbecco’s modified Eagle medium (DMEM; Life Technologies, Carlsbad, CA) containing 10% fetal bovine serum (FBS; Life Technologies), 100 U/mL penicillin, and 100 μg/mL streptomycin. Monocytes were infected with SFTSV at the indicated multiplicity of infection (MOI) at 37 °C. The inoculum was removed and the cells were washed three times with PBS before the cells were cultured in fresh DMEM supplemented with 2% FBS. The study was carried out by following the principles of the Declaration of Helsinki and the protocol approved by the Institutional Review Board of the College of Life Sciences, Nanjing University, in accordance with its guidelines for the protection of human subjects. The Institutional Review Board of the College of Life Sciences, Nanjing University, approved the collection of blood samples for the study, which was conducted in accordance with the guidelines for the protection of human subjects. Written informed consent was obtained from each participant.

### Ethics statement

C57BL/6 mice were provided by the Provincial Center for Disease Control and Prevention of Jiangsu. The animal study was approved by by the Ethics Committee of Jiangsu provincial CDC (Certificate No. JSCDCLL [2019]095), and was conducted in accordance with ARRIVE guidelines.

### Cells, reagents and viruses

Human embryonic kidney HEK293T cells from ATCC were grown in DMEM supplemented with 10% fetal bovine serum (Gibco, Carlsbad, CA), 1 mM sodium pyruvate (HyClone, Logan, UT), and 1% antibiotic–antimycotic solution (Gibco). The cells were cultured at 37 °C with 5% CO_2_. The SFTSV strain JS2014 was isolated by the Provincial Center for Disease Control and Prevention of Jiangsu and was used in this study^19^.

Glibenclamide and Ac-YVAD-cmk were obtained from InvivoGen Biotech (San Diego, CA). Monoclonal mouse anti-GAPDH (G9295) was purchased from Sigma-Aldrich (St Louis, MO). Monoclonal rabbit anti-NLRP3 (D2P5E), monoclonal rabbit anti-IL-1β (D3U3E), mouse IL-1β mAb (3A6), and monoclonal rabbit anti-caspase-1 (#4199) were purchased from Cell Signaling Technology (Beverly, MA). Monoclonal mouse anti-ASC (sc-271054) and polyclonal rabbit anti-IL-1β (sc-7884) were purchased from Santa Cruz Biotechnology (Santa Cruz, CA). Anti-AIM2 (ab93015), anti-NLRP1 (ab98181), and anti-NLRC4 (ab99860) antibodies were purchased from Abcam (Cambridge, UK). Rabbit polyclonal anti-Gasdermin D/DFNA5L (bs-14287R) antibody was from Bioss (Beijing, China).

### Enzyme-linked immunosorbent assay (ELISA)

Concentrations of human IL-1β and IL-18 in culture medium were measured by ELISA Kits from BD Biosciences (San Jose, CA). Mouse IL-1β, IL-18 and IL-6 ELISA Kits were purchased from the 4A Biotech (Beijing, China).

### Western blot analysis

Human PBMC whole-cell lysates were prepared by lysing the cultured cells with buffer (50 mM Tris–HCl, pH7.5, 300 mM NaCl, 1% Triton-X, 5 mM EDTA, and 10% glycerol) after culture medium was removed. The whole-cell lysates were clarified by low-speed centrifugation at 4 °C and the supernatants were measured for protein concentration using the Bradford assay (Bio-Rad, Hercules, CA). Supernatants (30 μg) were electrophoresed in an 8–12% SDS–polyacrylamide gel electrophoresis (PAGE) gel and the proteins were subsequently transferred to a polyvinylidene difluoride (PVDF) membrane (Millipore, MA). PVDF membranes were blocked with 5% skim milk in PBS with 0.1% Tween 20 (PBST) before they were incubated with antibody at an appropriate dilution. Protein signals on the membrane were detected using a Luminescent Image Analyzer (Fujifilm LAS-4000).

### Real-time polymerase chain reaction (RT-PCR)

Total RNA was extracted from human PBMCs with TRIzol reagent (Invitrogen) following the manufacturer’s instructions. Quantitative real-time PCR was performed with 1 μl of cDNA in a total volume of 10 μl with SYBR Premix Ex TaqII (Takara, Dalian, China) following the manufacturer’s instructions. Relative expression values were standardized to an internal GAPDH control. Real-time PCR primers were designed using Primer 3.0 (www.bioinfo.ut.ee/primer3) and sequences are provided in Supplementary Table S1.

Viral RNA was extracted from human blood samples, PBMCs, and mouse sera with the RNeasy kit (Qiagen, Germantown, MD). Real-time RT-PCR was performed using the QuantiTech RT-PCR kit (Qiagen) to measure viral RNA copy numbers for viral load. The primers were designed as previously described and used in a one-step real-time RT-PCR^20^. Amplification and detection were performed with an Applied Biosystems 7500 Real-time PCR system (Applied Biosystems, Foster City, CA). Data were analyzed using the software supplied by the manufacturer.

### Lentivirus production and transduction

The targeting sequences of shRNAs for human NLRP3, NLRP1, NLRC4, and AIM2 were as follows: sh-NLRP3: 5′-GGAGAGACCTTTATGAGAAAG-3′; sh-NLRP1: 5′-GCAAGCAATTAGAGCCTTTAG-3′; sh-NLRC4: 5′-GCTGTTCCAT ACCTTCTA TGA-3′ and sh-AIM2: 5′-GCCACTAAGTCAAGCTGAAAT-3′. The shRNA plasmids were transfected into HEK293T cells together with pMDLg, pRSV-rev, and pCMV-VSV-G using Lipofectamine 3000 (Life Technologies). The ratio of pLenti-sh NLRP3/NLRP1/NLRC4/AIM2, pMDLg, pRSVrev, and pCMV-VSV-G was 2:1:1:1, respectively. Culture supernatants were harvested at 24 and 48 h post-transfection (pt) and were centrifuged at 4500×*g* for 20 min at 4 °C to remove cellular debris. Recovered viruses were concentrated using an Amicon Ultra-15 centrifugal filter unit with an Ultracel-10 membrane (Millipore, Burlington, MA). The viruses were diluted in RPMI-1640 medium, and the viral titers were determined in HEK293T cells. Monocytes were transduced or infected with the same dose of the lentiviruses expressing scramble control or gene-specific shRNA in the presence of 8 μg/mL polybrene. After 48 h, monocytes were infected with SFTSV at various MOIs. Another 12 h later, the shRNA knockdown efficiency of the target protein in lentivirus-transduced cells was assessed by western blot analyses.

### Cell death assay

Cell death was assessed with EthD-III/calcein AM staining (Viability/Cytotoxicity Assay Kit, KeyGEN, China). While the calcein AM stain produced a bright green fluorescence in live cells, EthD-III entered dead cells and produced red fluorescence in dead cells. PBMCs, after various treatment and viral infection, were simultaneously stained with 2 mM calcein AM and 4 mM EthD-III for 30 min at room temperature. Samples were analyzed using flow cytometry (BDFACSVerse, BD Biosciences, San Diego). The percentage of dead cells was determined by counting the ratio of red-positive to blue-positive cells.

### Statistical analyses

Continuous variables were reported as means ± standard deviations (SD). Data were analyzed by SPSS 17.0 software. Group means were compared using one-way analysis of variance (ANOVA). Multigroup comparisons of the means were carried out by ANOVA test with post hoc contrasts by Student–Newman–Keuls (SNK) test. Pearson’s correlation tests were used to measure the strength of association between variables. Values of *P* < 0.05 were considered to be statistically significant.

## Results

### Increased levels of IL-1β were associated with disease severity and viral load in SFTS patients

**Figure 1:**
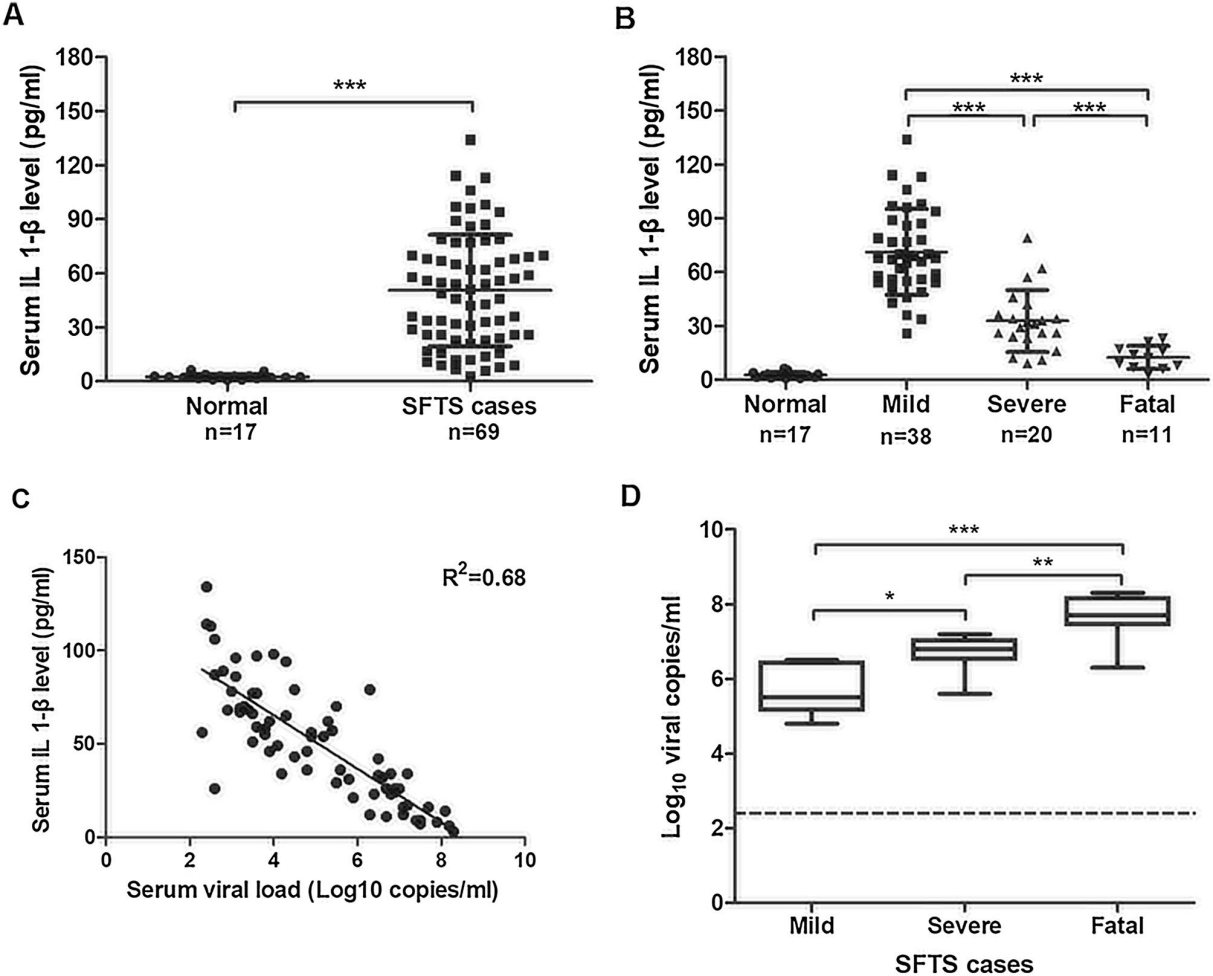
Elevated IL-1β secretion in blood of the SFTS patients. (**A**) Increased IL-1β levels in the sera of patients (n = 69) and healthy individuals (n = 17) was determined by ELISA. Data shown were means ± SEM; ***P < 0.001 (two-tailed Student's t-test). (**B**) IL-1β levels in the sera of the SFTS patients were negatively correlated to disease severity clinically. (**C**) Negative correlation was between the IL-1β levels and viral load in the sera of the SFTS patients. (**D**) The higher the serum virus titer in acute stage of SFTS cases, the more severe were the clinical symptoms. All serum samples were collected from SFTS patients on their second day of hospitalization. Viral load was determined based on the viral RNA copy numbers measured by RT-PCR with specific primers for the viral S genomic segment. R^2^ represented coefficient of the determination. r represented Pearson correlation coefficient, with r values of 0–0.3, 0.3–0.5 and > 0.5 indicating weak, moderate, and strong correlation, respectively.

SFTS is characterized by high fever and hemorrhage in patients, and these signs are persistent in severe cases. We hypothesize that these signs are associated with or caused by elevated proinflammatory cytokines such as IL-1β, which is processed by activated inflammasomes during the infection. To confirm that inflammasomes were activated in SFTS cases, we prepared serum samples from SFTS patients (n = 69), who were diagnosed clinically and confirmed with laboratory testing following the onset of disease, and analyzed the secretion levels of IL-1β in the sera by ELISA. Sera from healthy donors (n = 17) were prepared similarly and included as controls. The result showed that IL-1β levels in the sera of SFTSV-infected patients were significant higher than those in healthy individuals (Fig. 1A). We further assigned the samples from patients into one of three groups, mild, severe, and fatal, based on the severity of clinical disease. Our results showed that higher IL-1β levels were detected in the sera of acute phase patients who had relatively mild symptoms compared to the levels in the severely affected patients, and fatal patients had the lowest levels of IL-1β (Fig. 1B). The correlation between IL-1β secretion and viral load in serum was also evaluated and the results showed that IL-1β levels in sera were negatively associated with serum viral load (R^2^ = 0.68) (Fig. 1C). The higher the serum virus titer in acute stage SFTS cases, the more severe the clinical symptoms (Fig. 1D). Taken together, our data confirmed that proinflammatory IL-1β was induced and secreted in SFTS patients and its levels in sera were negatively associated with the severity of SFTS.

### SFTSV infection activated IL-1β production and secretion in monocytes

**Figure 2:**
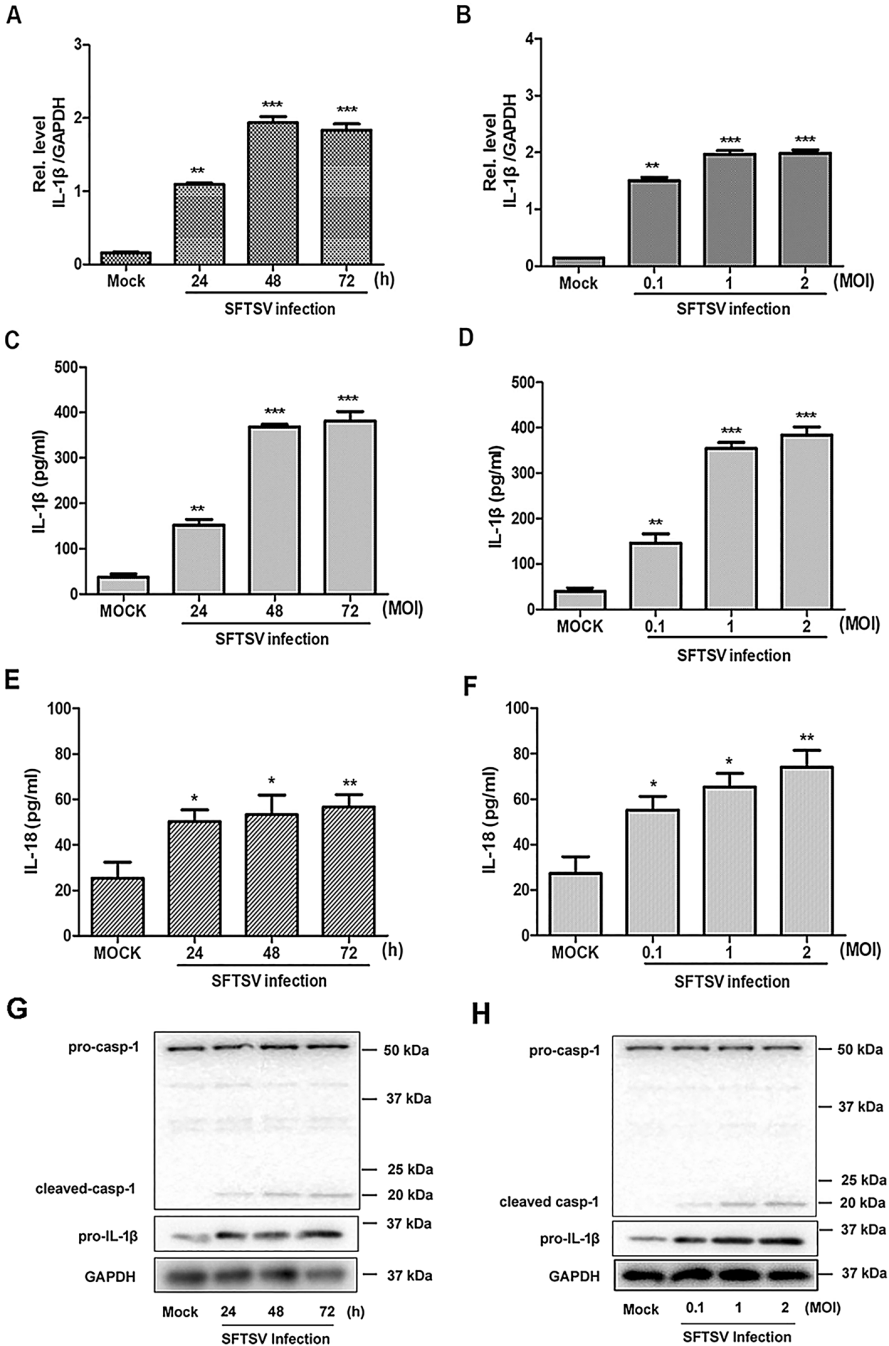
Induced upregulation or processing of pro-IL-1β, IL-18 and pro-caspase 1 in PBMCs infected with SFTSV. PBMCs were isolated from healthy individuals and infected with SFTSV at an MOI = 1 for 24, 48, or 72 h (**A**,**C**,**E**) or for 48 h at an MOI = 0.1, 1, or 2 (**B**,**D**,**F**). IL-1β and GAPDH mRNAs were quantified by RT-PCR with specific primers for IL-1β and GAPDH, respectively (**A**,**B**). IL-1β and IL-18 levels in the culture medium of PBMCs infected with SFTSV were determined by ELISA (**C**,**D**,**E**,**F**). Upregulation of pro-IL-1β and processing of cleaved caspase-1 (p20) were determined by western blot with specific antibodies for pro-IL-1β and cleaved caspase 1 (p20), respectively (**G**,**H**).

Various cell types had been found to be susceptible to SFTSV including monocytes^2,18,19,21^. The effect of SFTSV infection on pro-IL-1β induction and processing was determined in human PBMCs. We prepared human PBMCs from whole blood samples, which were infected with SFTSV. Our data showed that IL-1β mRNA transcripts were upregulated in PBMCs infected with SFTSV at various time points post infection (p.i.) (Fig. 2A) or at different multiplicities of infection (MOI) (Fig. 2B) as measured by RT-PCR. We further measured IL-1β and IL-18 secretion from PBMC cultures infected with SFTSV. The results showed that significant levels of secretedIL-1β and IL-18 was detected in culture medium at different time points and at different MOI p.i. (Fig. 2C–F).

We next examined pro-caspase-1 cleavage in PBMCs infected with SFTSV. As shown in Fig. 2G–H, both pro- and cleaved caspase-1 were detected and the cleaved caspase-1 increased in SFTSV-infected cells at different time points or MOI p.i. as determined by western blot analyses. Accordingly, the levels of cleaved IL-1β increased in the infected cells as well (Fig. 1S). This indicates that SFTSV infection induced an activation of inflammasomes, leading to pro-caspase-1 cleavage, IL-1β and IL-18 maturation and secretion in cultures of human PBMCs. PBMCs could be one of the key sources of the elevated IL-1β in the blood of SFTS patients.

### SFTSV infection induced inflammatory responses in mice

**Figure 3:**
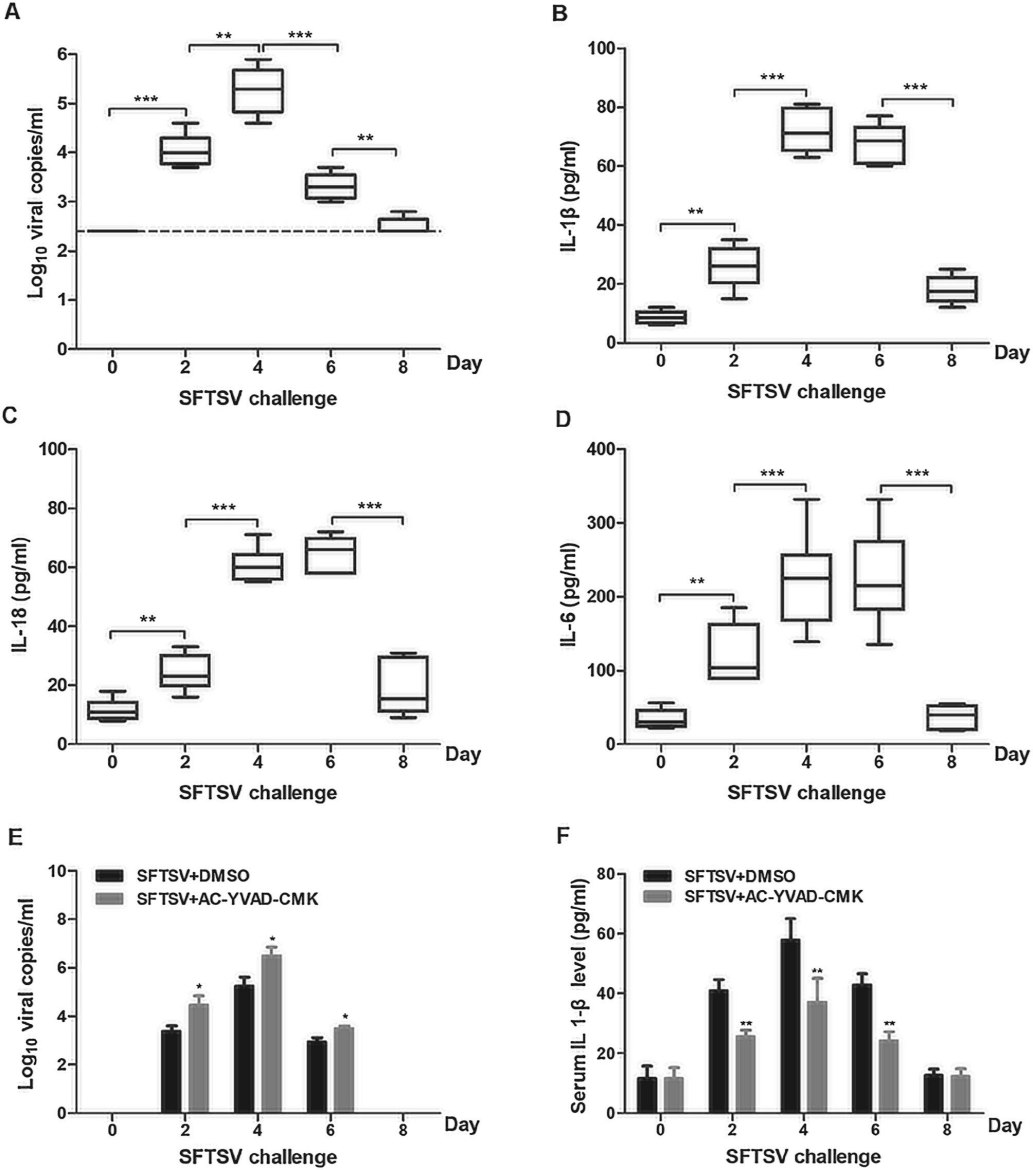
Caspase-1 dependent increase of IL-1β and IL-18 secretion in mice infected with SFTSV. Five-week-old C57BL/6 mice (male) were infected with SFTSV (1 × 10^5^ PFU) and measurement of IL-1β secretion and viral load in mouse sera was carried out at day 0, 2, 4, 6 and 8 p.i. Viral RNA copy numbers in the blood were determined by extraction of total RNA from the whole blood and RT-PCR with specific primers for the viral S genomic segment, while serum IL-1β levels by ELISA. (**A**) Viral RNA copy numbers and (**B**) IL-1β levels in the sera of the mice infected with SFTSV. C57BL/6 WT mice (5 weeks old, male) were pretreated with DMSO or Ac-YVAD-cmk (8 mg/kg) by intraperitoneal injection and then infected with SFTSV (1 × 10^5^ PFU) or with PBS. At each time point, on day 0, 2, 4, 6, and 8 days p.i., six mice from the Ac-YVAD-cmk or control group were sacrificed, and viral RNA copy numbers in the blood were determined by RT-PCR **(C)**. The levels of IL-1β, IL-18 and IL-6 in mouse sera were determined by ELISA (**D**–**F**). Data shown were whiskers: min.-max.; *P < 0.05, ***P < 0.01, ***P < 0.001 (one-way ANOVA with SNK post-hoc test).

The correlation between SFTSV infection and IL-1β/IL-18 secretion was evaluated in C57BL/6 mice. We inoculated individual mice with SFTSV at a dose of 5 × 10^5^ PFU/mouse, and blood samples were prepared at different days post inoculation. Infectious viral titers were determined in the sera of the inoculated mice, and showed that viral loads peaked at day 4 post inoculation and then declined (Fig. 3A). We also measured IL-1β and IL-18 levels in the sera of infected mice, which all increased rapidly until day 4 or 6and decreased thereafter (Fig. 3B,C). These data suggest that SFTSV infection can induce inflammasome activation with an associated increase in IL-1β/IL-18 production and secretion into the blood of the infected mice as well.

C57BL/6 mice were pre-treated with Ac-YVAD-cmk, (8 mg/kg) (n = 6), a caspase-1 inhibitor^22^, or DMSO (in control group) by intraperitoneal injection. Six hours later the mice were inoculated with SFTSV (5 × 10^5^ PFU/mouse). Serum samples were prepared from all inoculated mice and tested d for IL-1β. Our results showed that IL-1β was induced and secreted in the sera of the infected C57BL/6 mice, but levels of IL-1β in the sera were suppressed by inoculation of Ac-YVAD-cmk from day 2 through 6 (Fig. 3D), suggesting that the levels of IL-1β in mouse blood depends on caspase-1 activation for its secretion. To be noted, the virus was detected in the blood of infected mice but the infectious viral loads significantly increased in the mice treated with Ac-YVAD-cmk (Fig. 3E), suggesting that functional caspase-1 and IL-1β, or inflammasome activation, are associated with the protective process against SFTSV in infected mice.

### SFTSV activated the NLRP3 inflammasome to induce IL-1β secretion

**Figure 4:**
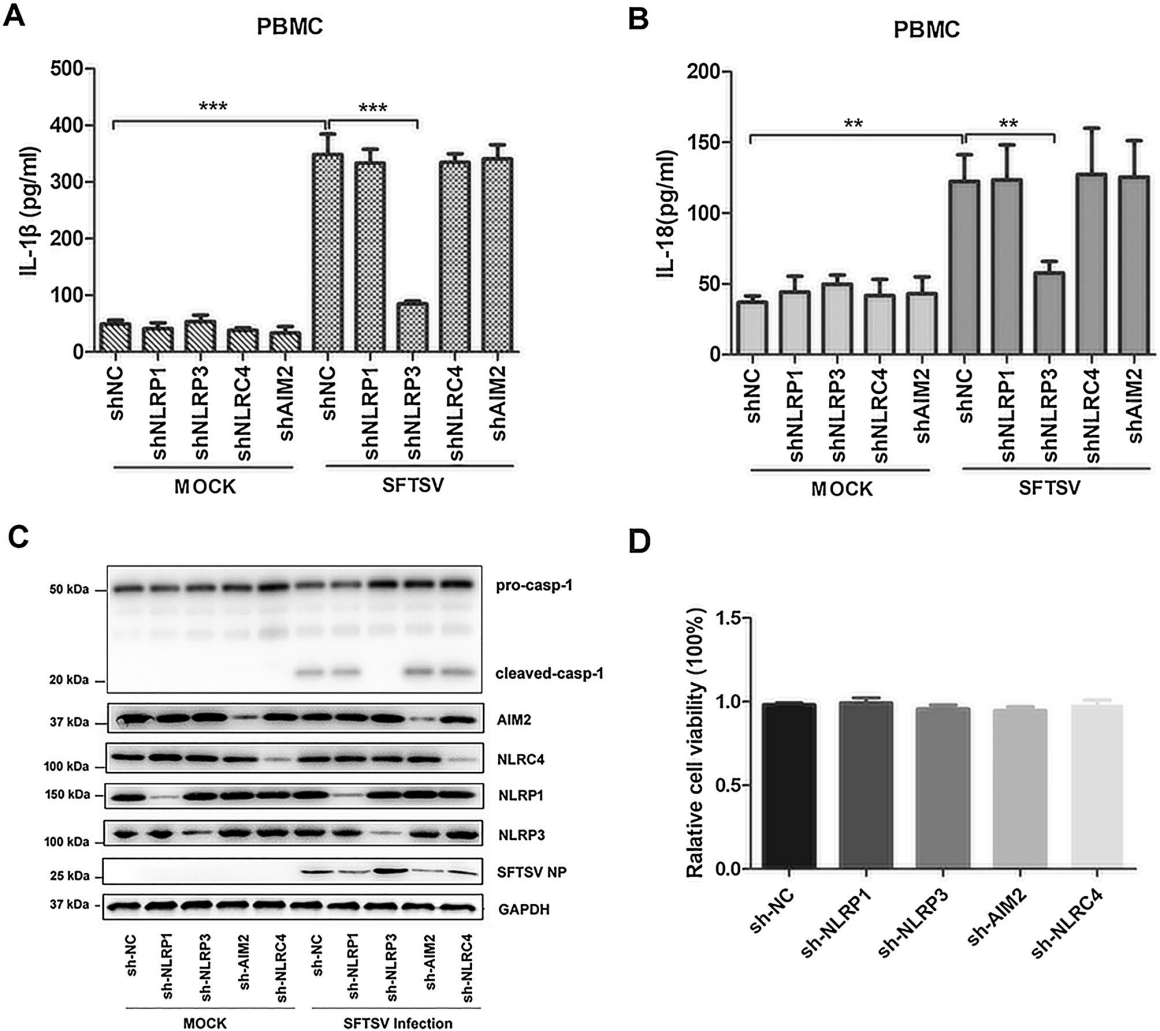
NLRP3 inflammasome dependent secretion of IL-1β and IL-18 in human PBMCs infected with SFTSV. Effect of NLR knockdown by shRNA on the processing of pro-caspase-1 and secretion of IL-1β was shown in PBMCs. The cells were infected with the lentiviruses which expressed shRNA specifically for knockdown of NLRP1, NLRP3, NLRC4 or AIM2, prior to be infected with SFTSV. (**A**,**B**) Secretion of IL-1β and IL-18 were measured in the culture medium of PBMCs infected with or without SFTSV by ELISA. (**C**) Knockdown of NLRs and cleavage of pro-caspase-1 in PBMCs infected with or without SFTSV detected by western blot with antibodies specific for respective proteins. (**D**) Comparable effect of shRNA on the viability of PBMCs as determined with a CCK-8 assay. ***P < 0.001. P-values were calculated using a One-way ANOVA test.

To characterize the mechanism of the inflammasome activation leading to maturation and secretion of IL-1β/IL-18 in SFTSV-infected patients, we examined the type of inflammasome activated in SFTSV-infected cells. Four shRNA cDNA molecules were designed and chemically synthesized to target genes of NLRP1, NLRP3, NLRC4, and AIM2, respectively. The cDNA of the shRNAs was cloned into a lentiviral vector, which yielded four recombinant lentiviral strains after plasmid transfection of packaging cells. PBMCs, prepared from healthy donor blood, were infected with the recombinant lentiviruses stably expressing shRNA targeting NLRP1, NLRP3, NLRC4, or AIM2, respectively, and detected for pro-IL-1β processing and secretion after the cells were infected with SFTSV. PBMCs infected with the lentivirus expressing scramble shRNA were used as control. As shown in Fig. 4A,B, elevated levels of IL-1β and IL-18 in the culture medium were detected in SFTSV-infected PBMCs expressing shRNA targeting NLRP1, NLRC4, and AIM2, respectively, comparable to the levels in the cells expressing scramble shRNA. However, the secretion of IL-1β and IL-18 was significantly lower in the SFTSV-infected cells expressing shRNA targeting NLRP3, as compared to the cells expressing shRNA targeting other types of NLRs as well as scramble shRNA.

We further examined the cleavage and processing of pro-caspase-1 and pro-IL-1β in the cells expressing shRNA targeting NLRP1, NLRP3, NLRC4, or AIM2 after SFTSV infection. As shown in Fig. 4C, while expression of NLRP1, NLRP3, NLRC4, or AIM2 was knocked down with corresponding shRNA, cleaved caspase-1 was significantly lower only in cells expressing shRNA targeting NLRP3 and not in the cells expressing shRNA targeting NLRP1, NLRC4, or AIM2. To be noted, the cell viability remained comparable in the cells expressing shRNA or scramble RNA as controls. This indicates that NLRP3 was required for the assembly of the functional inflammasome needed to cleave and activate pro-caspase-1, leading to maturation and secretion of IL-1β/IL-18 during SFTSV infection.

### SFTSV replication was inhibited by the NLRP3 inflammasome

**Figure 5:**
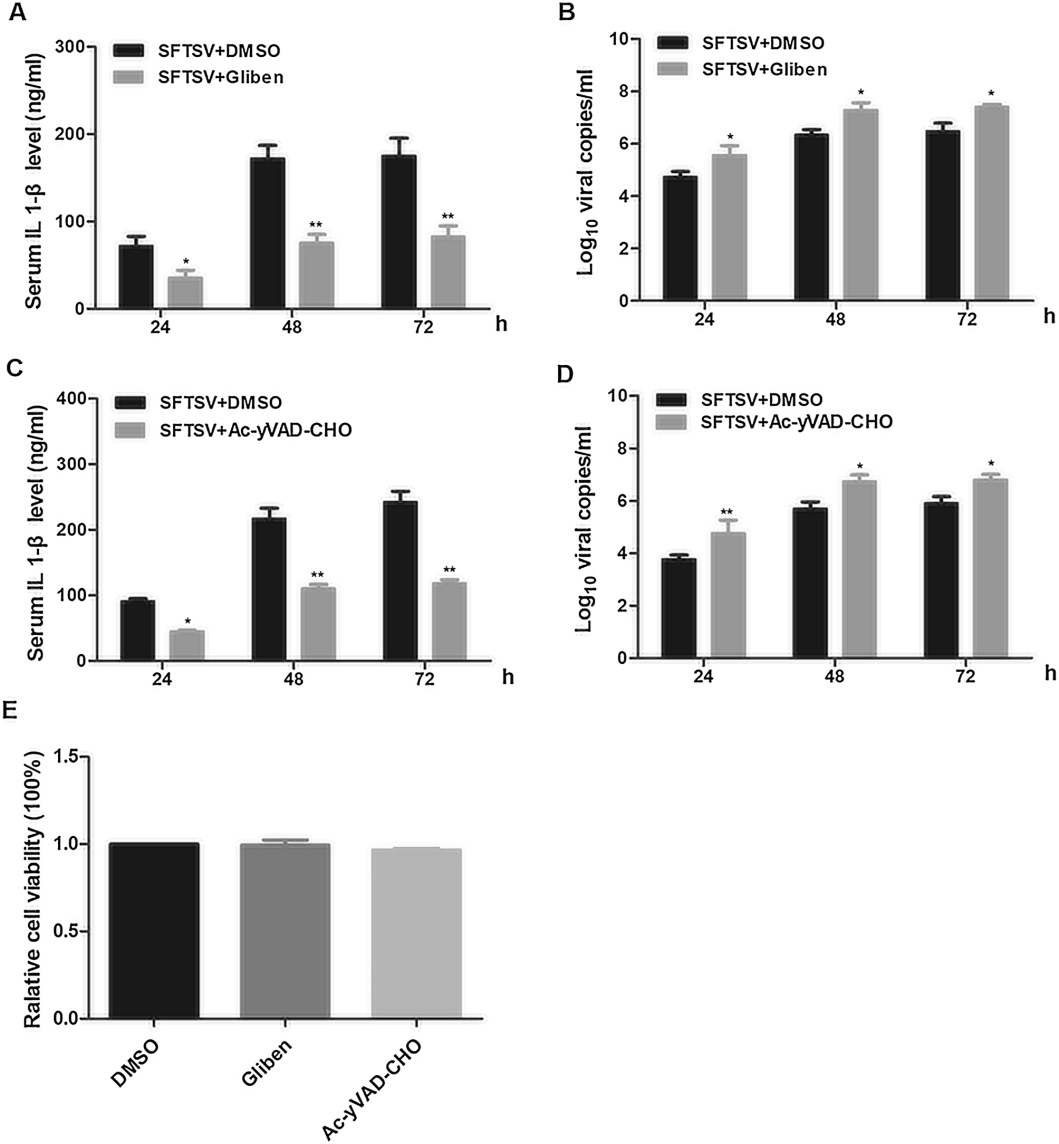
Effect of functional NLRP3 and caspase-1 on viral replication and IL-1β secretion in human PBMCs infected with SFTSV. Glibenclamide, an NLRP3-specific inhibitor, and Ac-YVAD-cmk, an inhibitor of caspase-1, were used to pre-treat PBMCs, prior to viral infection, to show their impact on IL-1β secretion and viral replication. (**A**,**B**) PBMCs were treated with glibenclamide (1 μM) or DMSO for 6 h, followed by infection with SFTSV (MOI = 1). IL-1β levels were determined in the culture medium at 24, 48, and 72 h pi by ELISA (**A**) and viral RNA copy numbers were measured in the total RNA extracted from the cells by real-time PCR with primers for the viral S genome (**B**). The (**C**,**D**) PBMCs were pre-treated with Ac-YVAD-cmk (1 μM) or DMSO for 6 h, followed by viral infection (MOI = 1). IL-1β levels were determined by ELISA (**C**) and the viral RNA copy numbers measured by real-time PCR (**D**). (**E**) No effect of glibenclamide (1 μM) or Ac-YVAD-cmk (1 μM) on liability of PBMCs were detected as determined with CCK-8 assays. The presented data were mean ± SD; n = 3; P-values were calculated using a One-way ANOVA test (*P < 0.05; **P < 0.01; ***P < 0.001).

To elucidate the role that NLRP3 inflammasome plays in viral replication in cells infected with SFTSV, PBMCs were pre-treated with glibenclamide, an NLRP3 inhibitor^23^, at 1 μM for 6 h, followed by infection of SFTSV at 1 MOI. Culture medium was sampled at different time points p.i. to test for secreted IL-1β and measure viral RNA copy numbers. As shown in Fig. 5A,B, the secretion of IL-1β decreased and the viral RNA copy numbers increased significantly in glibenclamide-treated cells in comparison to untreated cells, suggesting that a functional NLRP3 was required for IL-1β secretion and pyroptosis during SFTSV infection.

PBMCs, pre-treated with the caspase1-specific inhibitor Ac-YVAD-cmk at 1 μM for 6 h, were also infected with SFTSV at 1 MOI. IL-1β secretion and viral RNA copy numbersin the culture medium were determined as described. While IL-1β secretion decreased at 24, 48, and 72 h p.i., viral RNA copy numbers increased in the infected cells, compared to non-treated cells, shown in Fig. 5C,D, indicating that activity of the caspase-1 was essential to the processing and secretion of IL-1β.

We used a CCK-8 assay to determine the toxic effect of glibenclamide and Ac-YVAD-cmk on PBMCs at 1 μM for 6 h. Neither treatment at the indicated concentration had any significant effect on the viability of PBMCs (Fig. 5E).

### SFTSV infection induced pyroptosis and membrane damage of PBMCs

**Figure 6:**
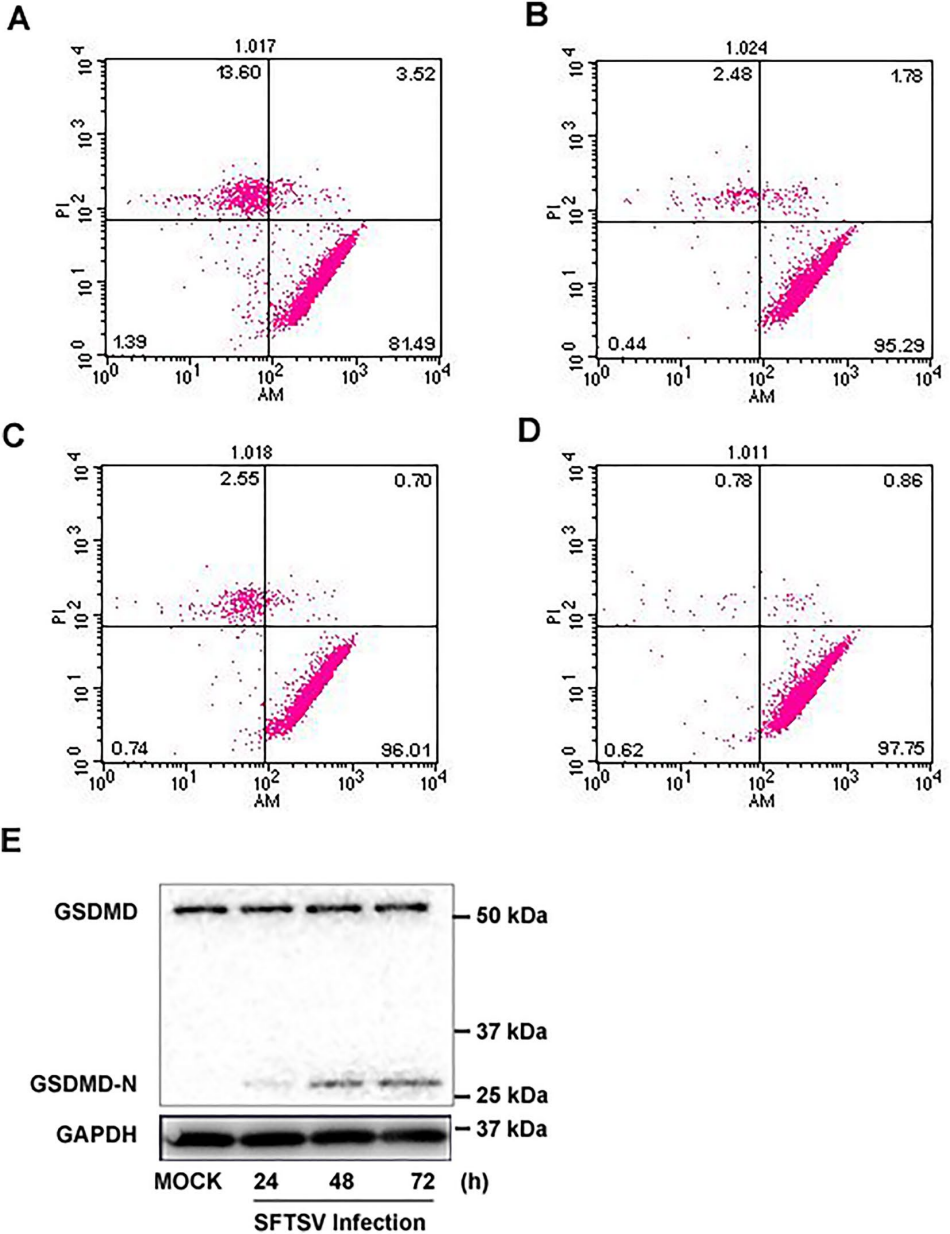
Inhibition of NLRP3 and caspase-1 reduced induction of membrane damage and cell death in PBMCs. PBMCs were pre-treated with DMSO alone, glibenclamide, or Ac-YVAD-cmk for 6 h prior to viral infection at an MOI of 1 for 48 h. The cells were subjected to staining with Calcein-AM/EthD-III for detection of membrane damage and cell death, which were analyzed by Flow cytometry. (**A**) DMSO alone plus SFTSV; (**B**) glibenclamide (1 μM) plus SFTSV; (**C**) Ac-YVAD-cmk (1 μM) plus SFTSV; and (**D**) DMSO. The ratio of membrane damage cells was determined using flow cytometry and differences between groups were analyzed by chi-square test. (**E**) Processing of GSDMD in PBMCs infected with SFTSV. The cells were with SFTSV an MOI of 1 and cell lysates were prepared at various time points p.i. for SDS-PAGE and western blot analysis with antibodies specific for GSDMD and GSDMD-N.

To determine whether cell death was involved in the death of monocytes induced by SFTSV infection, we infected PBMCs with SFTSV at an MOI of 1 and measured the membrane damage and cell death using the Calcein-AM/EthD-III Cell Activity Assay Kit at 48 h p.i. and the ratio of membrane damage cells was determined by flow cytometry. The results demonstrated that SFTSV infection induced cell death in infected PBMCs compared to mock-infected cells (Fig. 6A,B, *P* < 0.001). We also pre-treated PBMCs with 1 μM glibenclamide or Ac-YVAD-cmk, each for 6 h, followed by infection with SFTSV at an MOI of 1. While membrane damage was detected at 48 h p.i, we found that the proportion of dead cells with membrane damage was suppressed by either NLRP3 or caspase 1 inhibitors, the ratio of membrane damage cells decreased from 13.6 to 2.48% and 2.55% (Fig. 6B–D, P < 0.05).

We further examined PBMCs infected with SFTSV at an MOI of 1 and detected the expression of GSDMD and an increase of its cleaved form, GSDMD-N, after infection (Fig. 6E). Taken together, our data indicated that SFTSV infection induced inflammasome activation, leading to catalytic processing of GSDMD, production of the active GSDMD-N and pyroptosis, which contributed to death of infected PBMCs (Fig. 6E).

## Discussion

Inflammasome activation leading to the induction of host inflammatory responses was considered a critical mechanism of innate immunity in cells infected with microbes. Activation of inflammasomes is a complex process with multiple signaling pathways involved, including RIG-I-like receptors (RLRs), Toll-like receptors, and NF-κB signaling, leading to elevated expression of pro-IL-1β and pro-IL-18, processing and secretion of their mature forms, IL-1β and IL-18, and eventually pyroptosis^12,24^. IL-1β is a highly potent proinflammatory mediator that induces vasodilation and attracts granulocytes to inflamed tissues^12,16^. IL-1β is also an endogenous pyrogen prominent during the febrile phases of some viral infections and induces the production of prostaglandin E2 in the hypothalamus, resetting the hypothalamic thermostat to fever. In addition, IL-1β is associated with different clinical manifestations, such as coagulopathy and thrombocytopenia^25,26^. We have considered that IL-1β is also a key element in viral pathogenesis of SFTS which is an acute febrile disease with a tendency for hemorrhage. In this study, increased levels of IL-1β were confirmed in sera from acute phase SFTS cases. A transient increase of IL-1β was detected in a murine model infected with SFTSV. Monocytes were found to be susceptible to SFTSV infection and could be the key source of IL-1β secreted into blood, contributing to pathogenicity in infected hosts.

In addition to proinflammatory responses, the activation of inflammasomes and induction of pyroptosis could be part of the protective mechanisms directed at combatting viral infection^11,12^. Some RNA viruses are able to regulate the activation of inflammasome in order to counteract its effect during viral replication in host cells^12,27^. In this study, we found that in SFTS patients, higher IL-1β levels in sera of acute phase cases were correlated with relatively mild symptoms (Fig. 1B). Correlation between IL-1β levels and viral load in SFTS patients was also confirmed, which showed that IL-1β levels in acute phase sera were negatively correlated with serum viral load (R^2^ = 0.68) (Fig. 1C). It appeared when higher virus loads were in the sera of the acute stage, more severe the clinical symptoms ensued in SFTS cases (Fig. 1D). Reversal correlation of IL-1β levels and disease severity or viral loads in the acute phage of sera suggested that the process associated with increased IL-1β secretion could be protective against SFTSV, consistent with a previous report indicating that IL-1β could have dual roles in both the proinflammatory response and antimicrobial immunity in SFTSV infections^18^. Studies have shown that IL-1β can activate monocytes, macrophages and neutrophils, and is capable of driving the development of helper CD4 + T cells by regulating Th17 and Th1 responses^28,29^. Therefore, in hosts infected with SFTSV, activation of inflammasomes was not only an essential part of innate immunity in response to viral replication, but also a critical component of the adaptive immune responses for viral clearance.

Whereas IL-1β is a potent pro-inflammatory cytokine that is crucial for host-defense responses to infection, few data suggest a direct inhibitory effect of IL-1β on viral replication. Increased secretion of IL-1β enhances proinflammation in the site of infection, in which infection is contained and lymphocytes are activated through interaction with viral antigen-presentation cells for adaptive immunity. In IL-1β inhibitor-treated animals, if viral replication is affected, it is most likely due to IL-1β associated proinflammatory responses that indirectly lead to the change of infectious viral titers in treated animals. This would be the same as the study performed with the inhibitor of caspase-1, resulting in the reduction of cleaved IL-1β levels, and presented in this report (Fig. 3).

NLRP3 and AIM2-mediated inflammasomes are the most commonly activated complexes when some RNA viruses infect host cells. Previous studies showed that the NLRP3 inflammasome can be activated by infections of ZIKA virus, Foot-and-mouth disease virus, and Rift Valley Fever virus^22,30,31^, while the AIM2 inflammasome can be triggered by Chikungunya virus and West Nile Virus (WNV)^32^. ZIKV facilitates the NLRP3 inflammasome assembly and activation through viral NS5 protein interacting with the NACHT and LRR domains, which leads to the secretion of IL-1β and induction of an aggressive host inflammatory response closely related to viral pathogenicity^22^. Inflammasome activation has been identified in viral diseases which are common in febrile symptoms, indicating a significant advance in understanding their pathogenicity. In this study, SFTSV infection induced processing of pro-caspase-1 and subsequent maturation and secretion of IL-1β/IL-18 were significantly suppressed in human PBMCs when NLRP3 gene was knocked down by specific shRNA (Fig. 4) or inhibited by a specific inhibitor, glibenclamide (Fig. 5). Knockdown of several other types of NLRs did not have any inhibitory effect on the elevated IL-1β/IL-18 levels in SFTSV-infected PBMCs, demonstrating that SFTSV infection triggered IL-1β/IL-18 secretion by activating the NLRP3 inflammasome. We further examined the effect of the NLRP3 inflammasome activation on viral replication in SFTSV infected human PBMCs. Our data showed that viral RNA copies increased, or that viral replication was enhanced in PBMCs when there was an NLRP3 knockdown or inhibition by glibenclamide. We further demonstrated that effect of the NLRP3 inflammasome on viral replication in SFTSV-infected PBMCs and mice was caspase-1 dependent. When treated with caspase-1 inhibitor, the induction of IL-1β secretion in sera was suppressed and the viral replication was promoted (Fig. 3). Taken together, these data show that inflammasome activation was NLRP3 dependent and increased IL-1β secretion was essential for NLRP3 inflammasome function in SFSTV-infected human PBMCs and mice.

Activation of inflammasome can induce pyroptosis, a form of programmed cell death, through activation of pro-caspase-1 and subsequent cleavage of GSDMD^31,33,34^. Pyroptosis, as a form of inflammatory cell death dependent on caspase-1^34,35^, can have two important functions in protecting host cells against microbial infection. First, death of infected cells can lead to eradication of an intracellular pathogen and premature abortion of replicative process; Second, release of intracellular contents can enhance inflammation in infected tissues, distinct from anti-inflammatory apoptosis^36^. It is no surprise that pyroptosis has been reported to play an important role in inhibiting the replication of many viruses^23,31,35^. In this study, we observed that SFTSV infection induced pyroptosis with cleaved GSDMD in a proportion of PBMCs (Fig. 6) which could be attenuated by inhibition of NLRP3 or caspase-1 (Fig. 6C,D). Further, our data showed that, when inflammasome activation was suppressed by inhibiting caspase-1, viral replication was enhanced in infected PBMCs (Fig. 5), demonstrating that the induction of pyroptosis in monocytes might be contributing to protection against SFTSV by eliminating infected cells and/or enhancing local inflammatory responses in the host.

It is unclear what mechanism was involved in the NLRP3 inflammasome activation in SFTSV-infected PBMCs or monocytes. In a previous study Moriyama et al.^37,38^ reported that both wild-type NSs and its 21/23A mutant of SFTSV suppressed NLRP3 inflammasome-dependent IL-1β secretion in HEK293 cells transfected with plasmids expressing NLRP3 and SFTSV NSs. It remains to be characterized whether NSs can regulate the inflammasome activation in infected cells or animals and the mechanism could be variable in different tissue or cell types. On the other hand, NSs has been confirmed to be involved in dysregulating RIG-I signaling during SFTSV infection. In order to promote viral replication, SFTSV has developed various mechanisms to evade the host immune response through the interaction between NSs and RIG-I signaling components, including TBK1 and IRF3, sequestering them into the SFTSV-induced cytoplasmic structures as inclusion bodies (IBs)^38–40^. However, we were unable to identify the involvement of MAVS, a component of the mitochondria and key in RIG-I signaling, in the activation of the NLRP3 inflammasomes. As shown in our data, MAVS did not interact with NLRP3 in SFTSV-infected PBMCs (Fig. 2S), although this interaction did occur in the cells infected with other bunyavirus such as Rift Valley fever virus^41^.

We are aware that our study has limitations in several aspects. IL-1β may be secreted from monocytes, macrophages and many other cell types in the various tissues and organs of a host. We were unable to determine whether the IL-1β, detected in the sera, was solely secreted from the monocytes in SFTS patients, although monocytes were part of the PBMC culture studied in vitro in this report. Activation of NLRP3 or other NLR inflammasomes leading to IL-1β/IL-18 secretion and pyroptosis may also have different impact on viral infection and host cell status in different tissues and organs. Platelets could well be a source of IL-1β and may play an important role in the host inflammatory responses and viral pathogenicity in SFTSV-infected patients. We have preliminary data at this stage to indicate that platelets may also secrete IL-1β with pro-caspase-1 processed upon infection with SFTSV.

In conclusion, our report provides the evidence that SFTSV infection induced activation of the NLRP3 inflammasome, which led to elevated IL-1β/IL-18 secretion and pyroptosis in PBMCs and mice. Increased IL-1β and pyroptosis could be important in both pathogenicity and host protection against SFTSV as shown in infected PBMCs where viral replication was enhanced with knocked down or suppressed NLRP3 and caspase-1. Overall, our data support the notion that the NLRP3 inflammasome-dependent IL-1β secretion and pyroptosis characterized in this study may contribute to the viral pathogenesis in SFTSV infection. This study provides valuable insights for deciphering the mechanism about how this emerging phlebovirus causes a severe hemorrhagic fever in humans.

## Supplementary Information

Supplementary Information.

## Data Availability

All relevant data are within the manuscript and its Supporting Information files.
