# Supplementary material for: Activation of the NLRP3 inflammasome and elevation of interleukin-1β secretion in infection by sever fever with thrombocytopenia syndrome virus
Source: Sci Rep. 2022 Feb 16;12:2573. doi: 10.1038/s41598-022-06229-0 (PMC8850576; doi:10.1038/s41598-022-06229-0)

Supporting information

Activation of the NLRP3 Inflammasome and Elevation of Interleukin-1β Secretion in Infection by Sever Fever with Thrombocytopenia Syndrome Virus

Zhifeng Li[1, 2], Jianli Hu[2], Changjun Bao[2], Chengfeng, Gao[1], Nan Zhang[2], Carol J. Cardona[3], and Zheng Xing[3*]

1 Medical School and Jiangsu Provincial Key Laboratory of Medicine, Nanjing University, Nanjing, China

2 Jiangsu Provincial Center for Disease Prevention and Control, Nanjing, China

3 Department of Veterinary Biomedical Sciences, College of Veterinary Medicine, University of Minnesota at Twin Cities, Saint Paul, MN, USA

Table S1 Nucleotide sequences of primers and probes used in the this study

Genes	Primers	Sequences (5’-3’)
SFTSV	Forward Primer	TAAACTTCTGTCTTGCTGGCTCC
	Reverse Primer	TGGCAAGATGCCTTCACCA
	MGB Probe	CGCATCTTCACATTGAT
pro-IL-1β	Forward Primer	CACGATGCACCTGTACGATCA
	Reverse Primer	AGCACTGAAAGCATGA
GAPDH	Forward Primer	ACAGTCAGCCGCATCTTCTT
	Reverse Primer	ACGACCAAATCCGTTGACTC

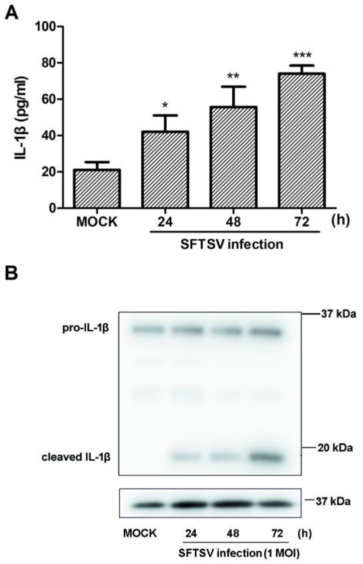

Figure S1. The production and secretion of IL-1β are induced by SFTSV in PBMCs. PBMCs were infected by the SFTSV (MOI = 1) for 24, 48 and 72 h. Supernatants were analyzed by ELISA for IL-1β secretion (**A**); Cell lysates were analyzed by western blot analysis using an antibody specific for IL-1β (**B**).

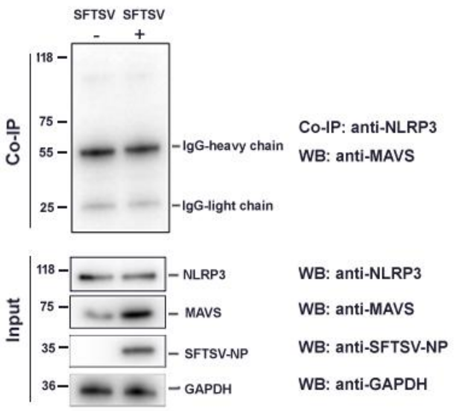

Figure S2. Interaction of NLRP3 and MAVS in PBMCs infected with SFTSV. Coimmunoprecipitation of NLRP3 and MAVS in SFTSV-infected PBMCs was shown. The cells were either mock-infected or infected with SFTSV for 48 hrs. The cell lysates were immunoprecipitated by an anti-NLRP3 antibody, and the immunoprecipitates were subjected to western blot analysis with an anti-MAVS antibody. The membrane was also blotted with anti-NLRP3, anti-MAVS and anti-SFTSV-NP antibodies as the input.

Figure S3. Original western blot images from figures used in this study

The blots were cut according to the molecular weight of the target antigens prior to hybridisation with antibodies during blotting. The edges of the blots were outlined using solid black lines.

Figure 2G

caspase1

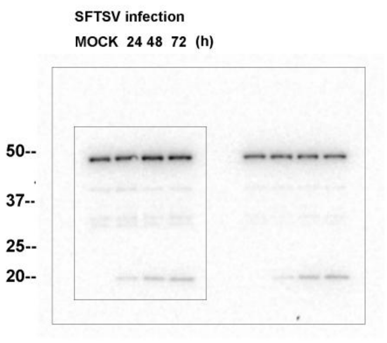

pro-IL-1β

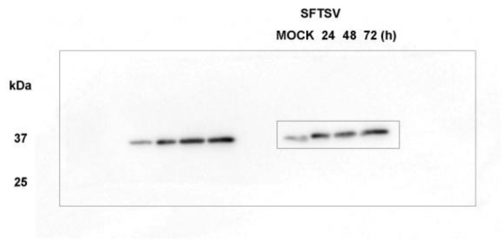

GAPDH

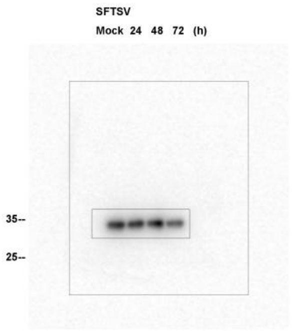

Figure 2H

caspase 1

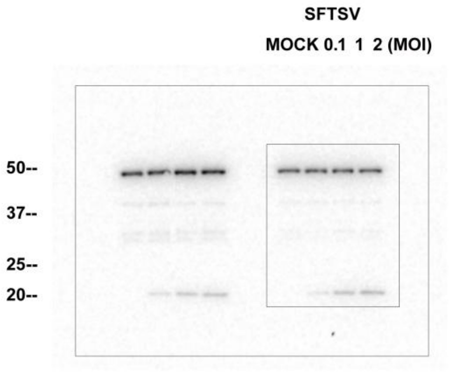

pro-IL-1β

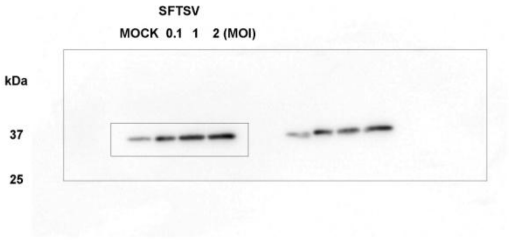

GAPDH

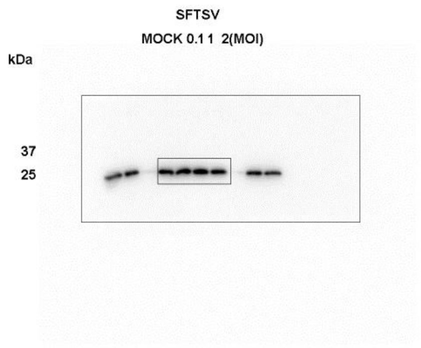

Figure 4C

Caspase-1

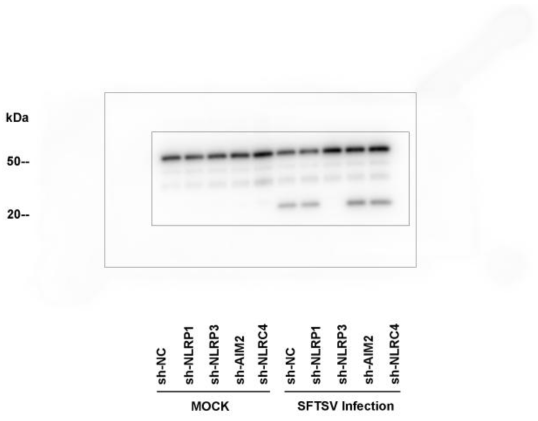

AIM-2

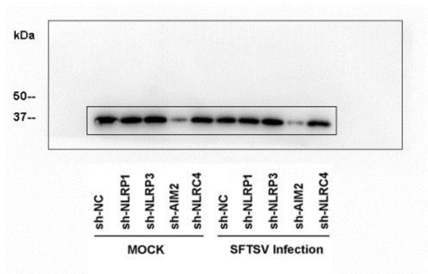

NLRC4

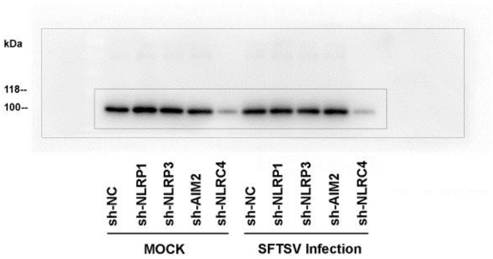

NLRP1

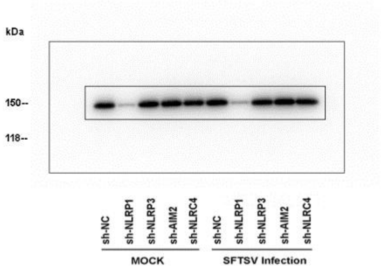

NLPR3

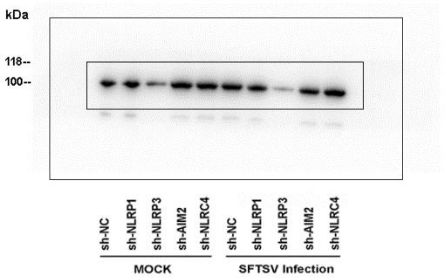

SFTSV-NP

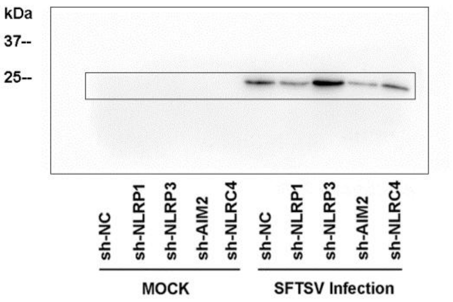

GAPDH

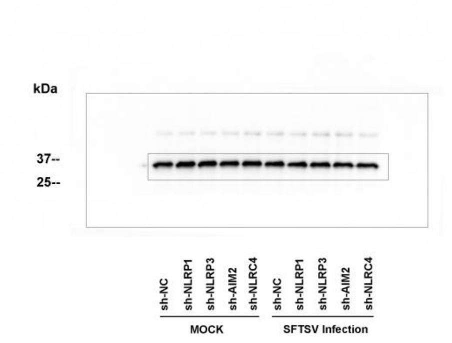

Figure 6E

GSDMD

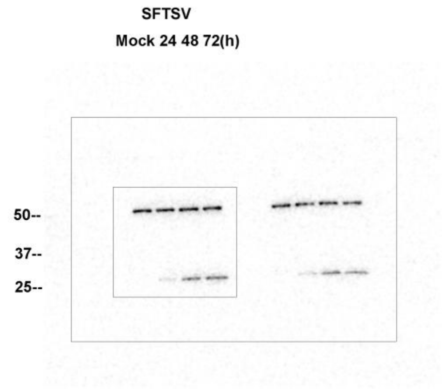

GAPDH

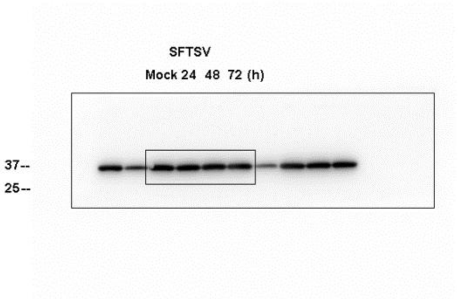

Figure S1B

IL-1β

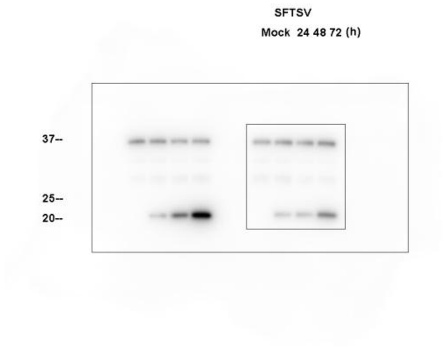

GAPDH

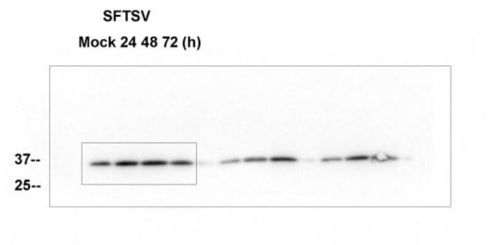

Figure S2

Heavy chain and light chain of IgG

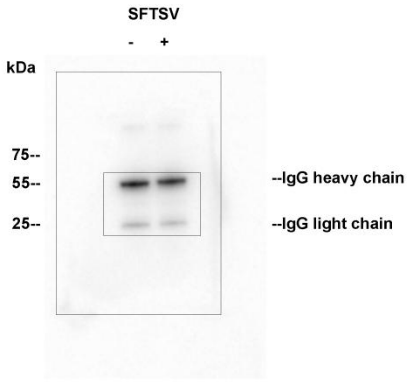

NLPR3

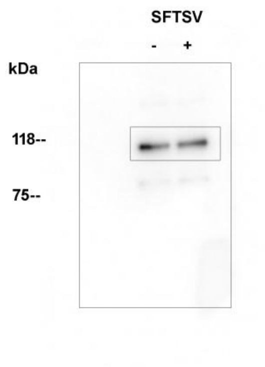

MAVS

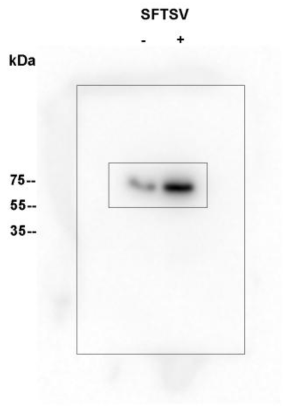

SFTSV-NP

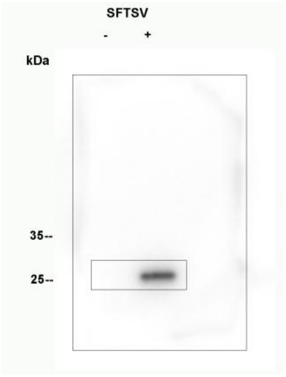

GAPDH

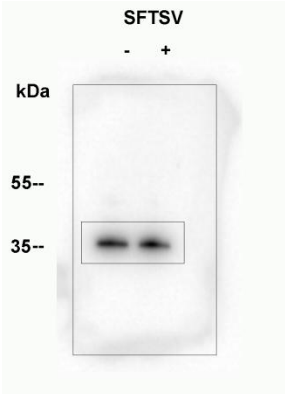

Supplement: Supplementary file 1 — Supplementary Information. [file 41598_2022_6229_MOESM1_ESM.pdf]
